# Nutritional Indices Are Associated with Mortality in the Elderly Patients Undergoing Left Atrial Appendage Occlusion: A Comparative Study of CONUT, GNRI, and PNI

**DOI:** 10.3390/jcdd13050177

**Published:** 2026-04-24

**Authors:** Ugur Karagoz, Enise Nur Ozlem Tiryaki, Enis Behcet Agirdici, Berke Ege, Muhammet Mucahit Tiryaki, Emre Ozdemir, Sadık Volkan Emren

**Affiliations:** 1 Department of Cardiology, Faculty of Medicine, Atatürk Research and Training Hospital, Katip Çelebi University, Izmir 35620, Türkiye; berke.ege@saglik.gov.tr (B.E.); emre.ozdemir1@ikcu.edu.tr (E.O.); sadikvolkan.emren@ikcu.edu.tr (S.V.E.); 2 Department of Neurology, Mus State Hospital, Mus 49200, Türkiye; enisenur.ozlem@saglik.gov.tr; 3 Department of Cardiology, Izmir City Hospital, Izmir 35540, Türkiye; e.agirdici@saglik.gov.tr; 4 Department of Cardiology, Mus State Hospital, Mus 49200, Türkiye; m.tiryaki@saglik.gov.tr

**Keywords:** malnutrition, controlling nutritional status, geriatric nutritional risk index, prognostic nutritional index, elderly

## Abstract

Background: We investigated the prognostic value of nutritional indices in patients with atrial fibrillation (AF) undergoing percutaneous left atrial appendage occlusion (LAAO). Methods: This two-center retrospective study enrolled 151 patients (median age 75, IQR: 69–80) undergoing LAAO. The Controlling Nutritional Status (CONUT) score, Geriatric Nutritional Risk Index (GNRI), and Prognostic Nutritional Index (PNI) were calculated preoperatively. Endpoints included all-cause mortality (primary), postoperative bleeding, and stroke. Associations with mortality were analyzed using multivariable Cox regression models. Results: Over a median follow-up of 8 months (IQR: 5–13), 28 patients (18.5%) died. In multivariable analyses (adjusted for age, sex, diabetes, and chronic kidney disease), each 1-point increase in the CONUT score was associated with a higher risk of all-cause mortality (HR 1.196, 95% CI 1.029–1.390; *p* = 0.020), whereas higher GNRI values were associated with a lower mortality risk (HR 0.956, 95% CI 0.915–0.998; *p* = 0.042). In contrast, PNI was not associated with mortality (*p* = 0.993). Nutritional indices did not significantly predict secondary outcomes like bleeding or stroke. Conclusions: These findings suggest that malnutrition is strongly and independently associated with mortality in high-risk AF patients receiving LAAO. The CONUT score demonstrates the most robust association in this population, highlighting the importance of metabolic reserves.

## 1. Introduction

Atrial fibrillation (AF) is the most common cardiac arrhythmia encountered in clinical practice. Ischemic stroke is considered one of the most severe complications associated with AF. Approximately 90% of thrombi originate within the left atrial appendage (LAA) [1]. Left atrial appendage occlusion (LAAO) is an appropriate intervention for protection against cardioembolic stroke in AF patients at increased risk of bleeding. Key guidelines and international consensus documents suggest LAAO as an indication in patients with contraindications to oral anticoagulant therapy requiring thromboembolic prophylaxis [2,3]. Nevertheless, LAAO is an invasive procedure and, although rare, complications include periprocedural stroke, pericardial effusion/tamponade, device embolization, vascular bleeding, and mortality. Such risks may be significantly mitigated through judicious patient selection, optimal imaging guidance (such as transesophageal echocardiography), extensive procedural experience, and individualized antithrombotic strategies [4].

Malnutrition has been recognized as a risk indicator for poor clinical outcomes in various diseases [5,6]. Previous studies have demonstrated that cardiovascular diseases remain the leading contributors to mortality among older adults, particularly in the presence of obesity-related metabolic vulnerability, underscoring the importance of early risk stratification in elderly cardiovascular populations [7]. While functional and composite indices like the Malnutrition-Inflammation Score (MIS) and handgrip strength are increasingly recognized as proximal to frailty, objective laboratory-based tools such as the Control of Nutritional Status (CONUT) score, Geriatric Nutritional Risk Index (GNRI), and Prognostic Nutritional Index (PNI) offer rapid, standardized, and easily calculable assessments in acute clinical settings [8]. Such multidimensional indices provide a better reflection of a patient’s overall metabolic and inflammatory status than isolated biochemical parameters [9]. Consequently, nutritional evaluation tools have been demonstrated to have prognostic worth in patients with malignancy, coronary artery disease, heart failure, and peripheral arterial disease [10,11,12,13]. Extending beyond these structural and ischemic conditions, malnutrition—when evaluated by these specific scoring systems—has also been reported as an independent estimator of all-cause mortality in patients with non-valvular AF [14]. Therefore, nutritional status assessment provides useful insight for evaluating prognostic associations and stratifying risk in non-valvular AF patients.

LAAO candidates represent a unique group characterized by advanced age, greater frailty, and a higher bleeding risk compared to the general AF population [15]. In this vulnerable group, physiological reserve, as reflected by nutritional status, may be as critical a determinant of survival as procedural factors. Our study aimed to evaluate the relationship between nutritional scores and clinical outcomes in patients with atrial fibrillation who had undergone left atrial appendage occlusion.

## 2. Materials and Methods

### 2.1. Patient Population

This was a two-center retrospective cohort study including consecutive patients who underwent percutaneous LAAO between January 2020 and July 2025 at the Cardiology Clinics of Izmir Kâtip Çelebi University Atatürk Training and Research Hospital and Izmir City Hospital. Patients were excluded if they met any of the following criteria: (i) presence of moderate-to-severe mitral stenosis or a mechanical prosthetic valve; (ii) acute systemic infection or sepsis within 30 days prior to the procedure, which could transiently confound baseline nutritional parameters; (iii) advanced hepatic failure (Child-Pugh Class C) or end-stage renal disease (estimated glomerular filtration rate < 15 mL/min/1.73 m^2^); and (iv) missing baseline laboratory data required to calculate the nutritional indices or a complete lack of follow-up survival data. Patients were selected based on guideline-directed heart team decisions. The term ‘elderly’ was used descriptively for this cohort, given the advanced median age typical of LAAO candidates. Overall, 151 patients were ultimately included in the study. During the initial retrospective screening phase, patients were excluded strictly due to missing essential baseline laboratory parameters (such as albumin or total lymphocyte count) or being completely lost to follow-up immediately post-discharge.

### 2.2. Data Collection

The demographic and clinical parameters of the participants were retrieved from the electronic medical database. Specifically, detailed data regarding the patients’ past medical history and baseline comorbidities—including hypertension, diabetes mellitus, coronary artery disease, congestive heart failure, chronic kidney disease, and prior history of stroke, transient ischemic attack, or major bleeding—were systematically extracted based on established physician diagnoses and admission notes at the time of the LAAO procedure. All biochemical measurements were obtained from tests performed within 24 h before the LAAO procedure. Complete blood count (neutrophils, lymphocytes, platelets, and hemoglobin) and biochemical test (serum urea, creatinine, albumin, cholesterol) parameters were recorded. Transthoracic and transesophageal echocardiography were acquired 24 h prior to the procedure. Laboratory assays were standardized between the two participating centers. These measurements were performed in accredited central laboratories using automated analyzers subjected to routine internal and external quality control protocols. Left ventricular ejection fraction (EF) was measured using the bi-plane Simpson method. The presence of left atrial appendage thrombus and spontaneous echo contrast (SEC) in the left atrium was also noted. Procedural details, including the specific types of LAAO devices utilized—namely the Amplatzer™ Amulet™ (Abbott Structural Heart, Plymouth, MN, USA) and the Lifetech LAmbre™ (Lifetech Scientific, Shenzhen, China)—were obtained from catheterization laboratory reports. The antiplatelet and anticoagulant medications used by the patient before and after the procedure were documented.

The primary endpoint was the mortality rate from all causes, which was followed up through phone interviews and medical record reviews. Final survival status was defined within the study cohort in October 2025. The observation period concluded on 31 October 2025, or at the time of patient death, whichever occurred first.

### 2.3. Nutritional Status

CONUT is based upon three laboratory variables: serum albumin (g/dL), total lymphocyte count (/mm^3^), and total cholesterol (mg/dL). Scoring for each variable is as follows: albumin ≥ 3.50 = 0; 3.00–3.49 = 2; 2.50–2.99 = 4; <2.50 = 6. Lymphocytes ≥ 1600 = 0; 1200–1599 = 1; 800–1199 = 2; <800 = 3. Total cholesterol ≥ 180 = 0; 140–179 = 1; 100–139 = 2; <100 = 3. Total (0–12 range) 0–1 is classified as normal, 2–4 as mild, 5–8 as moderate, and 9–12 as severe malnutrition.

Geriatric Nutrition Risk Index (GNRI). GNRI was calculated as GNRI = 14.89 × albumin (g/dL) + 41.7 × (actual weight/ideal weight). The weight/ideal weight ratio was limited to 1.0 when >1.0, and the ideal weight was obtained using the Lorentz formula. The GNRI is classified as follows: >98 no risk, 92–98 low risk, 82–91 moderate risk, and <82 high risk.

Prognostic Nutrition Index (PNI). PNI is calculated as PNI = 10 × albumin (g/dL) + 0.005 × lymphocyte count (/mm^3^). PNI is categorized as >45 normal/low risk, 40–45 moderate risk, and <40 high risk.

### 2.4. Statistical Analysis

Statistical analyses were performed using R version 4.4.0 (R Foundation for Statistical Computing, Vienna, Austria). Continuous variables are presented as median (interquartile range [IQR]) or mean ± standard deviation, while categorical variables are presented as frequencies and percentages. For the primary time-to-event analysis of all-cause mortality, follow-up time was calculated from the date of LAAO to death or censoring at the end of follow-up. Survival curves were generated using the Kaplan–Meier method and compared with the log-rank test. Associations between nutritional indices (CONUT, GNRI, and PNI) and all-cause mortality were evaluated using Cox proportional hazards regression. Patients with missing baseline laboratory or follow-up data were excluded from the primary analysis, and complete-case analysis was utilized for all regression models. Nutritional indices were analyzed primarily as continuous variables; additional Kaplan–Meier analyses were performed using predefined risk categories. A prespecified multivariable clinical model including age, sex, diabetes mellitus, and chronic kidney disease was used as the baseline model, and each nutritional index was added separately to assess incremental prognostic value. These baseline covariates were selected a priori due to their established prognostic relevance and strong clinical association with mortality in elderly cardiovascular populations. Results are reported as hazard ratios (HR) with 95% confidence intervals (CI). The proportional hazards assumption was evaluated using Schoenfeld residuals. Median follow-up was summarized using the reverse Kaplan–Meier method. Model fit and discrimination were assessed using Akaike’s Information Criterion (AIC) and Harrell’s concordance index (C-index), respectively. To reduce optimism in model discrimination estimates, internal validation was performed using bootstrap resampling to obtain optimism-corrected C-index values. In addition, receiver operating characteristic (ROC) curve analyses were performed for 12-month mortality to determine the optimal cut-off values for CONUT and GNRI. Optimal thresholds were identified using the Youden index, and the corresponding area under the curve (AUC), sensitivity, and specificity values were calculated. Given the low number of post-procedural cerebrovascular events and major bleeding events, associations between nutritional score categories and these secondary outcomes were assessed using Fisher’s exact test and were considered exploratory. All statistical tests were two-sided, and a *p*-value < 0.05 was considered statistically significant.

## 3. Results

**Figure A1:**
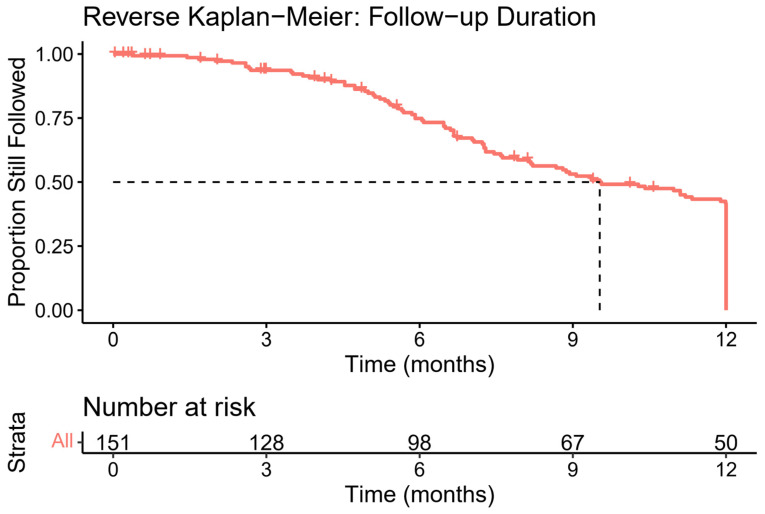
Reverse Kaplan–Meier curve illustrating follow-up duration and censoring patterns. The curve represents the proportion of patients still being followed at each time point, with a median follow-up of 9.5 months. Censoring events include both mortality and loss to follow-up.

In our study, a total of 151 patients who underwent LAAO were evaluated. One hundred and twenty-three (81.5%) of them survived, while 28 (18.5%) died. The median follow-up period was 8 months (IQR 5–13). The distribution of follow-up duration in the population was analyzed using the inverse Kaplan–Meier method and is displayed in Appendix A, Figure A1.

Table 1 presents the baseline clinical, echocardiographic, and laboratory characteristics of the overall study population (*n* = 151). The median age of the cohort was 75 years (IQR: 69–80), and 52.3% of the patients were male. The study population was characterized by a high burden of comorbidities, including hypertension (74.8%), coronary artery disease (49.0%), and diabetes mellitus (45.7%). The median LVEF was 60% (IQR: 50–60). Baseline nutritional assessment revealed a median CONUT score of 2 (IQR: 1–4), a median GNRI of 96.8 (IQR: 90.8–99.8), and a median PNI of 44.2 (IQR: 40.3–48.7), demonstrating varying degrees of nutritional risk prior to the procedure. Regarding procedural characteristics, the Amplatzer™ Amulet™ was successfully implanted in 89 patients (58.9%), while the LAmbre™ device was utilized in 62 patients (41.1%).

**Figure A2:**
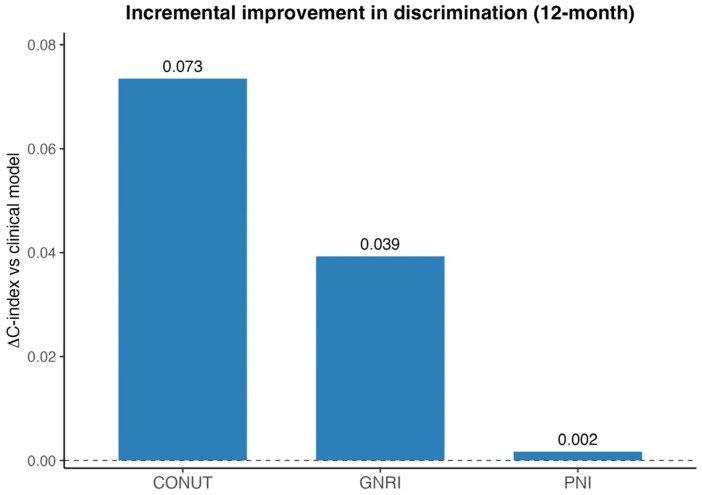
Incremental improvement in model discrimination with the addition of nutrition scores to the baseline clinical model. C-index: Concordance index.

In the primary time-to-event analysis using multivariable Cox proportional hazards regression, each nutritional index was added separately to a prespecified baseline clinical model including age, sex, diabetes mellitus, and chronic kidney disease (Table 2). In the baseline model, none of the clinical covariates reached statistical significance. Adding CONUT score was associated with a 19.6% higher mortality risk per 1-point increase (HR 1.196, 95% CI 1.029–1.390; *p* = 0.020) and improved model performance (AIC 243.6 → 240.7; C-index 0.621 → 0.695). Higher GNRI values were associated with lower mortality risk (HR 0.956, 95% CI 0.915–0.998; *p* = 0.042) with a modest improvement in model fit/discrimination (AIC 241.5; C-index 0.661). Adding PNI did not provide additional prognostic value (HR 1.000, 95% CI 0.974–1.027; *p* = 0.993; AIC 245.6; C-index 0.623). In bootstrap internal validation, optimism-corrected C-index values were 0.570 for the clinical model, 0.631 for the model including CONUT, 0.604 for the model including GNRI, and 0.548 for the model including PNI. The corresponding 12-month change in discrimination (ΔC-index) is illustrated in Appendix A, Figure A2. ROC analysis for 12-month mortality identified an optimal cut-off value of 4.5 for CONUT, with an AUC of 0.650, sensitivity of 44.0%, and specificity of 82.5%. For GNRI, the optimal cut-off value was 96.05, with an AUC of 0.656, sensitivity of 72.0%, and specificity of 57.9%.

The relationship between secondary outcomes and nutritional scores is shown in Table 3. Because there were very few post-procedural cerebrovascular events and major bleeds, we approached these analyses as exploratory and used descriptive categorical comparisons instead of formal time-to-event models. Based on Fisher’s exact test, which was chosen due to the low number of events, there was no significant link between nutritional score categories and post-procedural CVE or major bleeding.

**Figure 1:**
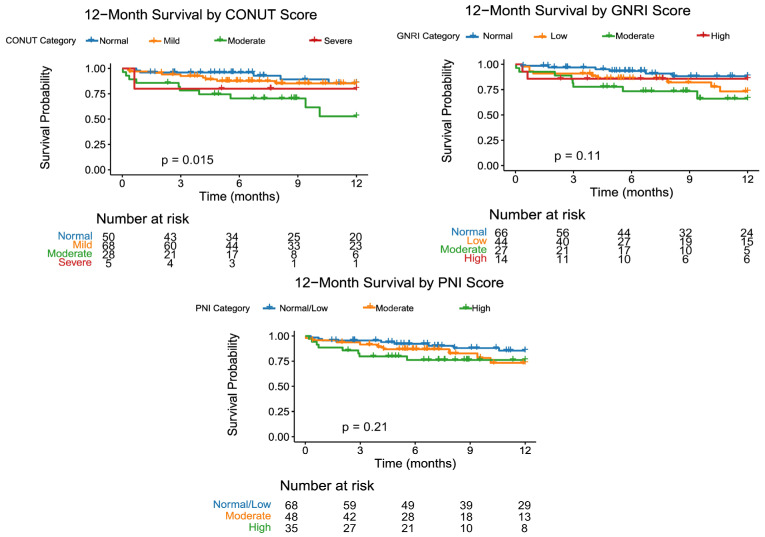
Survival analysis according to CONUT score categories. The log-rank test was used for comparison between groups.

In the 12-month Kaplan–Meier survival analysis (Figure 1), the curves separated significantly among CONUT categories; the normal and mild groups demonstrated similar and higher survival rates, while survival was lower, particularly in the moderate malnutrition group (log-rank *p* = 0.015). Although survival curves for GNRI showed a tendency toward better outcomes as scores increased, this difference was not found to be statistically significant (*p* = 0.11). Although the curve for the high-risk group in PNI was lower, the total separation was not statistically significant (*p* = 0.21).

## 4. Discussion

In our study, the prognostic influence of nutritional indices measured before the procedure on clinical outcomes in patients receiving LAAO was comprehensively assessed. The major finding of our study, derived from time-to-event survival analyses, is that malnutrition, determined by CONUT and GNRI scores, is strongly and independently associated with an increased hazard of all-cause mortality after LAAO. This finding suggests that nutritional depletion reflects reduced physiological reserves and systemic illness. However, adding nutritional scores to the baseline clinical model shows that malnutrition has independent prognostic value beyond that of advanced age or comorbidity burden.

Left atrial appendage closure provides an alternative treatment choice, particularly for atrial fibrillation patients who are contraindicated for OAC use or who have a high bleeding risk. Unfortunately, these patients are often elderly, have multiple comorbidities, and are frail [16]. Even if the LAAO procedure is performed well, the patient’s mortality risk remains high due to reduced physiological reserve. CONUT, GNRI, and PNI scores are crucial parameters that shed light on the estimation of this physiological reserve in the practice setting. The use of nutritional status indices for prognostic risk determination in this high-risk population may be reasonable. Studies in AF patients have reported that malnutrition, defined by nutritional scores, is associated with all-cause mortality [14,17]. Recent literature has begun to explore the outcomes experienced by malnourished patients following LAAO, thereby highlighting the clinical relevance of this procedure. Our study builds on this by providing a comparative assessment of multiple nutritional indices in order to identify the most clinically effective tool [18].

CONUT and GNRI scores emerged as the leading prognostic factors in our study. Even after adjusting for known prognostic factors, including age, gender, diabetes mellitus, and chronic kidney disease, an incremental 1-point increase in the CONUT score and a decrease in the GNRI score were both significantly associated with an increased risk of mortality. Although univariable ROC analyses showed comparable absolute AUC values for CONUT and GNRI, multivariable analysis demonstrated that CONUT provides a substantially greater incremental prognostic value when added to standard clinical risk factors. As described in the method, the CONUT score includes serum albumin, total lymphocyte count, and total cholesterol levels, whereas the GNRI is based on serum albumin and body weight/ideal weight ratio. Therefore, the superior prognostic power of CONUT and GNRI in our cohort likely stems from their comprehensive ability to capture systemic metabolic depletion, specifically including low cholesterol in the CONUT score and low body mass in the GNRI, in addition to hypoalbuminemia. The link between low total cholesterol and low HDL cholesterol levels and increased mortality can be attributed to the “cholesterol paradox” in the LAAO population. Given that our study cohort is an elderly group with multiple comorbidities, low cholesterol levels should be interpreted as an indicator of increased inflammation, cachexia, and serious systemic disease instead of atherosclerotic protection [19]. The CONUT score integrates this paradox into its prognostic model by scoring low cholesterol as a risk factor. Similarly, the inclusion of body weight in the GNRI serves as a marker of sarcopenia and frailty, which are often underdiagnosed in LAAO patients. A low GNRI reflects diminished somatic protein and muscle mass, which directly affects recovery capacity and physiological resilience following an invasive procedure [20].

One of the other findings of our study is that the PNI, which is frequently used in the literature, is not a statistically significant predictor of mortality in multivariable analysis, unlike CONUT and GNRI. This discrepancy arises because the PNI formula relies solely on serum albumin concentrations and total lymphocyte counts, disregarding systemic metabolic and somatic parameters. Thus, the isolated evaluation of albumin and lymphocytes by the PNI may not adequately capture the severe metabolic and somatic depletion seen in this specific elderly LAAO cohort, rendering it less predictive than the multi-dimensional CONUT or GNRI scores. This suggests that the key pathophysiological factors determining mortality in this specific LAAO population are metabolic (cholesterol) and somatic (body mass) depletion states, which are better reflected by CONUT and GNRI, rather than the immune-inflammatory condition indicated by lymphopenia, which is the focus of PNI. Therefore, PNI has been inadequate in addressing the risk profile of this specific cohort. There is evidence in the literature supporting the notion that PNI has less prognostic power compared to CONUT and GNRI; for example, in the AFTER-2 study involving 2592 non-valvular AF patients, CONUT and GNRI scores were strongly predictive of mortality in these patients, while the PNI score was found to have no statistically significant influence on survival [21].

Kaplan–Meier survival analysis through 12 months in our study provides a visual confirmation of the prognostic value of categorical nutritional scores. The analysis of CONUT scores demonstrated that, especially in the moderate and severe malnutrition groups, survival was significantly and markedly worse. On the other hand, no significant differences were observed between groups for GNRI and PNI scores. The discordance detected for GNRI between the Kaplan–Meier and Cox model results is noteworthy. Grouping patients for categorical analysis distributed the event count, decreasing the statistical power to detect differences between cohorts. Furthermore, we must consider the possibility of reverse causation, whereby patients in a progressively worse state of health develop malnutrition as a consequence of their severe underlying cardiovascular status. Continuous variable analysis, however, had higher statistical power, and the relationship between GNRI and mortality reached statistical significance in the Cox model.

We assessed the effect of nutritional scores on morbidity through secondary endpoints, in addition to mortality as the primary endpoint. However, no significant relationship was observed between nutritional scores and postoperative bleeding or cerebrovascular events. While previous evidence-based studies have demonstrated that the CONUT score can predict stroke and bleeding in the general AF population, potentially due to malnutrition-induced systemic inflammation, endothelial dysfunction, and coagulopathy [22], our study did not observe this association. The lack of statistical significance regarding the secondary endpoints should not be interpreted as an absolute absence of biological association, as these acute and periprocedural events may be more strongly influenced by acute factors such as procedural technique, device-related thrombosis, or periprocedural antithrombotic regimen rather than the patient’s chronic nutritional status [23]. Furthermore, optimizing procedural techniques through appropriate multimodal imaging assessment is considered essential for mitigating periprocedural risks and improving overall outcomes. Advanced imaging modalities, including three-dimensional transesophageal echocardiography, intracardiac echocardiography, and contrast-enhanced computed tomography, have been shown to play a pivotal role in accurate anatomical evaluation, precise device sizing, and real-time procedural guidance. It is emphasized that the integration of these emerging imaging technologies significantly enhances patient selection, procedural success, and the long-term efficacy of the device [24]. This technical optimization is thought to synergize with clinical risk stratification tools, such as nutritional indices, to provide a comprehensive approach for managing high-risk LAAO candidates.

Our study has some limitations that are worth noting. First, given its two-center, observational, and retrospective design, this research can only demonstrate an association rather than a relationship of causation. This retrospective, criteria-based study design may introduce selection and collider biases. Furthermore, despite rigorous multivariable adjustment, residual confounding factors from unmeasured clinical or physiological variables cannot be completely eliminated. Given these methodological constraints, the generalizability of the findings to different patient populations and other centers may be limited. Second, our study has a relatively small sample size (n = 151) and a low event count for the primary endpoint (n = 28). In addition, no formal a priori sample size calculation was performed because of the retrospective design, and the relatively limited number of mortality events required a parsimonious modeling strategy to reduce the risk of overfitting. Consequently, the statistical power of the study may be limited, particularly for secondary endpoints and categorical analyses, and may have prevented some true relationships (e.g., trends in Kaplan–Meier for GNRI or PNI) from achieving statistical significance. Moreover, the low event rate relative to the number of baseline covariates may introduce statistical model instability. As a result, the regression results should be interpreted as strong associations rather than definitive predictive models. Nevertheless, the significant improvement in model discrimination demonstrates that the relationship between CONUT/GNRI and mortality represents a robust biological effect rather than statistical noise. Finally, our median follow-up period of 8 months is relatively short for evaluating long-term outcomes after LAAO. Longer follow-up studies are needed to investigate the long-term prognostic associations of nutritional status.

## 5. Conclusions

Our study demonstrates through time-to-event analysis that malnutrition is independently associated with all-cause mortality in high-risk AF patients undergoing percutaneous LAAO procedures. Among these markers, the CONUT score displayed the strongest association with mortality, while the GNRI also emerged as a significant risk marker. These scores, which can be simply calculated from routine laboratory and anthropometric data, allow for the detection of malnutrition status in LAAO candidates. Future prospective interventional studies are warranted to establish whether optimizing nutritional status prior to the procedure can modify risk and definitively improve clinical outcomes.

## Figures and Tables

**Table 1:** Baseline characteristics of the patients.

Variables	Overall
	*n* = 151
Age (years), median (IQR)	75 (69–80)
Sex (male), *n* (%)	79 (52.3)
Height (cm), median (IQR)	165 (160–170)
Weight (kg), median (IQR)	75 (65.5–85)
BMI, median (IQR)	26.7 (24.5–30.6)
HT, *n* (%)	113 (74.8)
DM, *n* (%)	69 (45.7)
CRF, *n* (%)	37 (24.5)
CAD, *n* (%)	74 (49.0)
CVD, *n* (%)	91 (60.3)
Bleeding, *n* (%)	
None	74 (49.0)
Minor	29 (19.2)
Major	48 (31.8)
LVEF (%), median (IQR)	60 (50–60)
LAA Thrombus, *n* (%)	15 (9.9)
LAA SEC, *n* (%)	35 (23.2)
BUN (mg/dL), median (IQR)	20 (16–26)
Creatinine (mg/dL), median (IQR)	1.1 (0.9–1.4)
eGFR (mL/min/1.73 m^2^), median (IQR)	54 (43.5–60)
Lymphocyte count (/mm^3^), median (IQR)	1530 (1175–2015)
Neutrophil count (/mm^3^), median (IQR)	4410 (3190–5725)
PLT (×10^3^/µL), median (IQR)	231 (179–288)
Hb (g/dL), mean (SD)	11.63 (2.01)
TG (mg/dL), median (IQR)	114 (79–162)
LDL Cholesterol (mg/dL), median (IQR)	99 (79–125.5)
HDL Cholesterol (mg/dL), median (IQR)	44 (36–50)
Total Cholesterol (mg/dL), median (IQR)	165 (138–201)
Albumin (g/dL), median (IQR)	3.7 (3.3–3.9)
ASA, *n* (%)	92 (61)
Clopidogrel, *n* (%)	122 (80.8)
Rivaroxaban, *n* (%)	
None	129 (85.4)
15 mg	4 (2.6)
20 mg	18 (11.9)
Edoxaban, *n* (%)	
None	144 (95.4)
60 mg	7 (4.6)
Apixaban, *n* (%)	
None	127 (84.1)
2.5 mg	3 (2.0)
5 mg	21 (13.9)
CONUT score, median (IQR)	2 (1–4)
GNRI score, median (IQR)	96.8 (90.8–99.8)
PNI score, median (IQR)	44.2 (40.3–48.7)
CONUT group, *n* (%)	
Mild	68 (45.0)
Normal	50 (33.1)
Moderate	28 (18.5)
Severe	5 (3.3)
GNRI group, *n* (%)	
None	66 (43.7)
Mild risk	44 (29.1)
Moderate risk	27 (17.9)
High risk	14 (9.3)
PNI group, *n* (%)	
Normal/Low risk	68 (45.0)
Moderate risk	48 (31.8)
High risk	35 (23.2)
Device type, *n* (%)	
Amplatzer Amulet	89 (58.9)
LAmbre	62 (41.1)
Post-procedural CVE	8 (5.3)
Post-procedural bleeding	
None	132 (87.4)
Minor	14 (9.3)
Major	5 (3.3)

ASA: Acetylsalicylic acid, BMI: Body mass index, BUN: Blood urea nitrogen, CAD: Coronary artery disease, CONUT: Controlling Nutritional Status, CRF: Chronic renal failure, CVD: Cerebrovascular disease, DM: Diabetes mellitus, eGFR: Estimated glomerular filtration rate, GNRI: Geriatric Nutritional Risk Index, Hb: Hemoglobin, HDL: High-density lipoprotein, HT: Hypertension, IQR: Interquartile range, LAA: Left atrial appendage, LDL: Low-density lipoprotein, LVEF: Left ventricular ejection fraction, PLT: Platelet count, PNI: Prognostic Nutritional Index, SEC: Spontaneous echo contrast, SD: Standard deviation, CVE: Cerebrovascular event, TG: Triglycerides.

**Table 2:** Incremental Cox regression models for all-cause mortality.

Hazard Ratios with 95% Confidence Intervals and *p*-Values				
Variable	Multivariable Cox Regression Models			
Model	Model 1: Clinical ^a^	Model 2: + CONUT	Model 3: + GNRI	Model 4: + PNI
Age (per year)	1.013 (0.964–1.064) *p* = 0.620	1.010 (0.962–1.060) *p* = 0.686	1.017 (0.968–1.068) *p* = 0.507	1.013 (0.963–1.065) *p* = 0.628
Male sex	1.877 (0.831–4.241) *p* = 0.130	1.907 (0.846–4.298) *p* = 0.119	2.045 (0.896–4.667) *p* = 0.089	1.877 (0.829–4.249) *p* = 0.131
Diabetes Mellitus	2.066 (0.879–4.860) *p* = 0.096	1.991 (0.817–4.467) *p* = 0.135	1.951 (0.827–4.602) *p* = 0.127	2.066 (0.878–4.861) *p* = 0.096
CKD	1.853 (0.811–4.237) *p* = 0.144	1.564 (0.667–3.665) *p* = 0.303	1.660 (0.711–3.878) *p* = 0.241	1.854 (0.809–4.245) *p* = 0.144
CONUT score	Reference	1.196 (1.029–1.390) * *p* = 0.020	Reference	Reference
GNRI score	Reference	Reference	0.956 (0.915–0.998) * *p* = 0.042	Reference
PNI score	Reference	Reference	Reference	1.000 (0.974–1.027) *p* = 0.993
AIC ^b^	243.6	240.7	241.5	245.6
C-index ^c^	0.621	0.695	0.661	0.623

* *p* < 0.05. ^a^ Model 1: Adjusted for age, sex, diabetes mellitus, and chronic kidney disease. ^b^ AIC: Akaike Information Criterion (lower values indicate better fit). ^c^ C-index: Concordance index (higher values indicate better discrimination). Optimism-corrected C-index (bootstrap): (Model 1: 0.570, Model 2: 0.631, Model 3: 0.604, Model 4: 0.548).

**Table 3:** Secondary Outcomes and Nutritional Scores.

Nutritional Score Category	Post-Procedural CVE (Fisher *p*)	Major Post-Procedural Bleeding (Fisher *p*)
CONUT category	0.625	0.758
GNRI category	0.546	0.404
PNI category	0.902	0.442

Categories were defined as described in the Nutritional Status section. CONUT: Controlling Nutritional Status; CVE: Cerebrovascular event; GNRI: Geriatric Nutritional Risk Index; PNI: Prognostic Nutritional Index.

## Data Availability

The data from this study are available upon request from the corresponding author.

## Abbreviations

The following abbreviations are used in this manuscript:

LAAO	Left Atrial Appendage Occlusion
AF	Atrial Fibrillation
CONUT	Controlling Nutritional Status
GNRI	Geriatric Nutritional Risk Index
PNI	Prognostic Nutritional Index
CKD	Chronic Kidney Disease
TEE	Transesophageal Echocardiography
TTE	Transthoracic Echocardiography
OAC	Oral Anticoagulation
